# Supplementary material for: A breakthrough series collaborative to increase patient participation with hemodialysis tasks: A stepped wedge cluster randomised controlled trial
Source: PLoS One. 2021 Jul 20;16(7):e0253966. doi: 10.1371/journal.pone.0253966 (PMC8291659; doi:10.1371/journal.pone.0253966)

**S1 Figure - Sequence of Learning Events and Action Period Calls.**

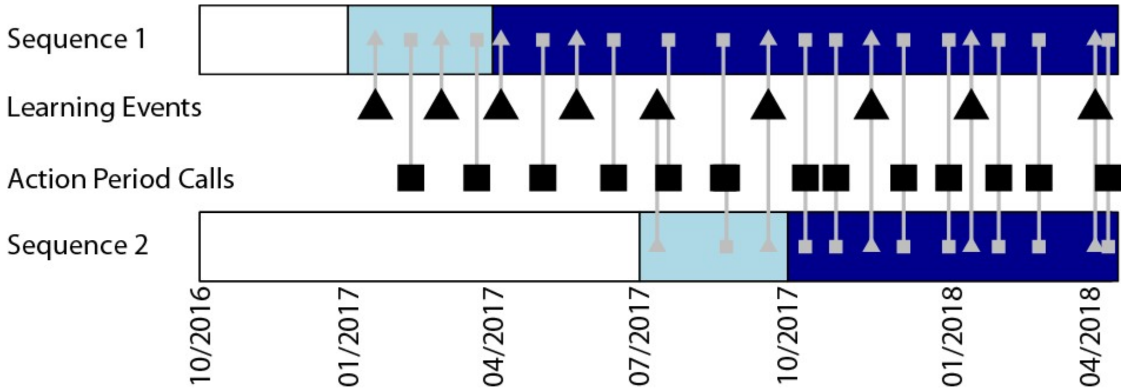

Supplement: S1 Fig — (PDF) [file pone.0253966.s002.pdf]
