# Supplementary material for: A breakthrough series collaborative to increase patient participation with hemodialysis tasks: A stepped wedge cluster randomised controlled trial
Source: PLoS One. 2021 Jul 20;16(7):e0253966. doi: 10.1371/journal.pone.0253966 (PMC8291659; doi:10.1371/journal.pone.0253966)
Supplement: S3 Fig — (PDF) [file pone.0253966.s004.pdf]

S5 Figure - Patient participation in individual dialysis tasks at baseline and end of the stepped wedge randomised controlled trial in patients doing 5 or more tasks at baseline. Categories: independent (blue), supervised (green) and not doing (red).

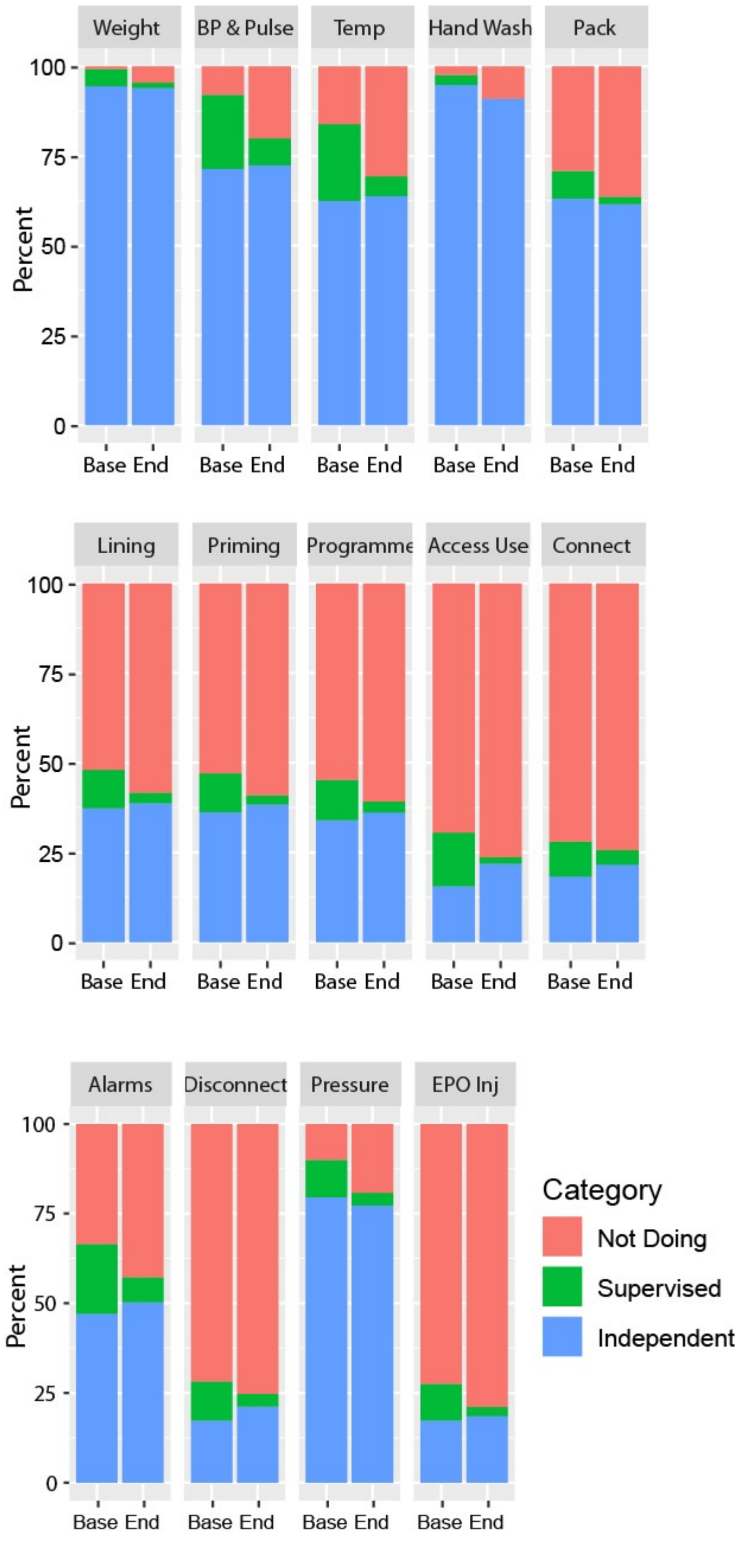

A BREAKTHROUGH SERIES COLLABORATIVE TO INCREASE PARTICIPATION WITH TREATMENT RELATED TASKS IN CENTRE-BASED HAEMODIALYSIS PATIENTS – A STEPPED WEDGE CLUSTER RANDOMISED CONTROLLED TRIAL
