# Supplementary material for: A breakthrough series collaborative to increase patient participation with hemodialysis tasks: A stepped wedge cluster randomised controlled trial
Source: PLoS One. 2021 Jul 20;16(7):e0253966. doi: 10.1371/journal.pone.0253966 (PMC8291659; doi:10.1371/journal.pone.0253966)
Supplement: S4 Fig — (PDF) [file pone.0253966.s005.pdf]

S6 Figure - Secular trends in secondary endpoints, stratified by randomisation sequence (1: Early, 2: Late): A) number of independent or supervised tasks over time, B) number of independent or supervised tasks over time stratified by baseline number of tasks, C) EQ5D Utility value over time, D) EQ5D Utility Value stratified by baseline number of tasks, E) Patient activation measure over time, F) Patient activation measure over time stratified by baseline Patient Activation level.

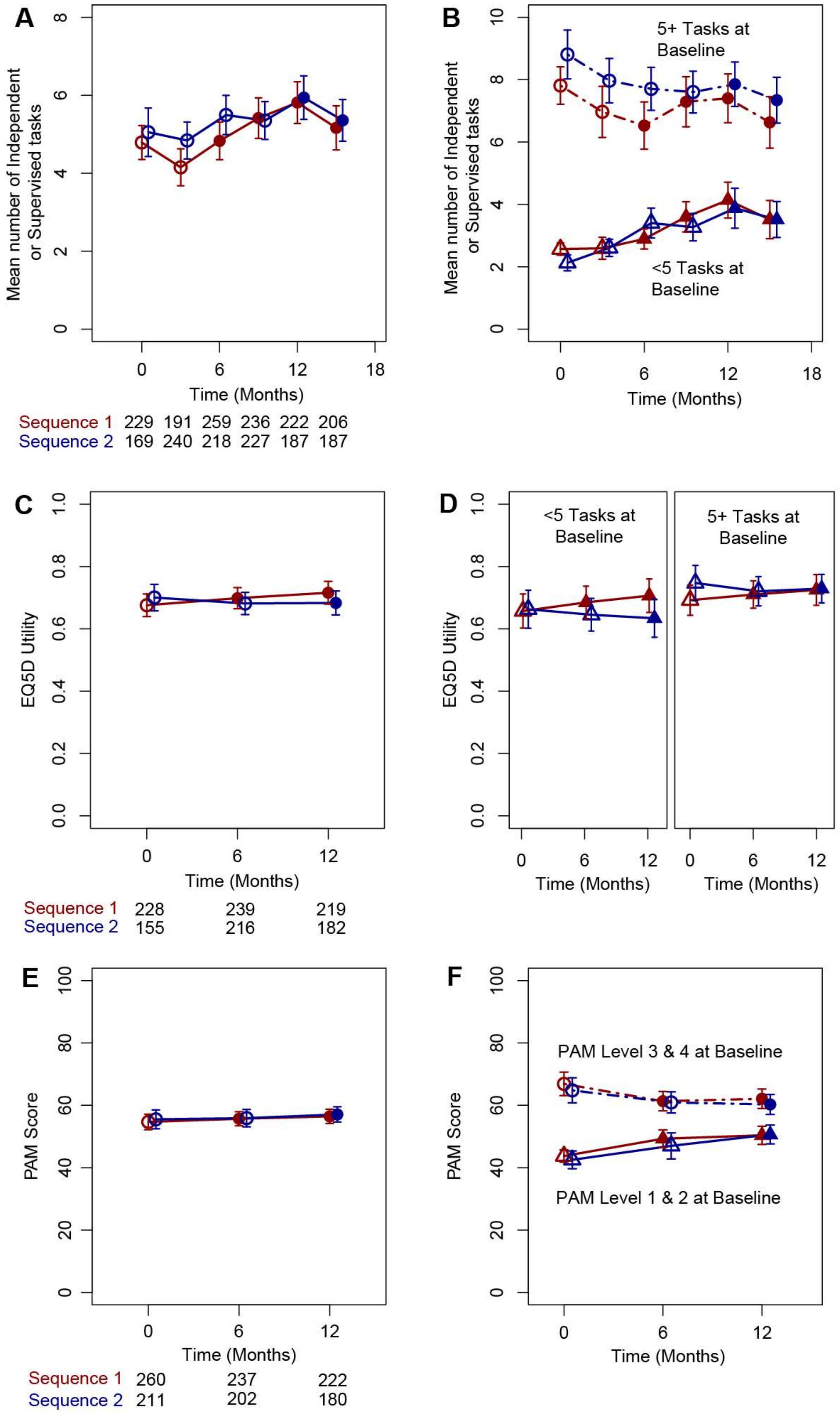

A BREAKTHROUGH SERIES COLLABORATIVE TO INCREASE PARTICIPATION WITH TREATMENT RELATED TASKS IN CENTRE-BASED HAEMODIALYSIS PATIENTS – A STEPPED WEDGE CLUSTER RANDOMISED CONTROLLED TRIAL
