# Supplementary material for: A breakthrough series collaborative to increase patient participation with hemodialysis tasks: A stepped wedge cluster randomised controlled trial
Source: PLoS One. 2021 Jul 20;16(7):e0253966. doi: 10.1371/journal.pone.0253966 (PMC8291659; doi:10.1371/journal.pone.0253966)

S12 Figure **The secular trends in A) hospitalisation (green, n=983) and emergency room attendance (red, n=1,123) and B) Cause specific hospitalisation: Infection – red (n=191), Vascular Access – green (n=106), Fluid Overload – blue (n=72) during the course of the stepped wedge randomised control trial. Lighter continuous lines represent sequence one and darker interrupted lines represent sequence 2.**

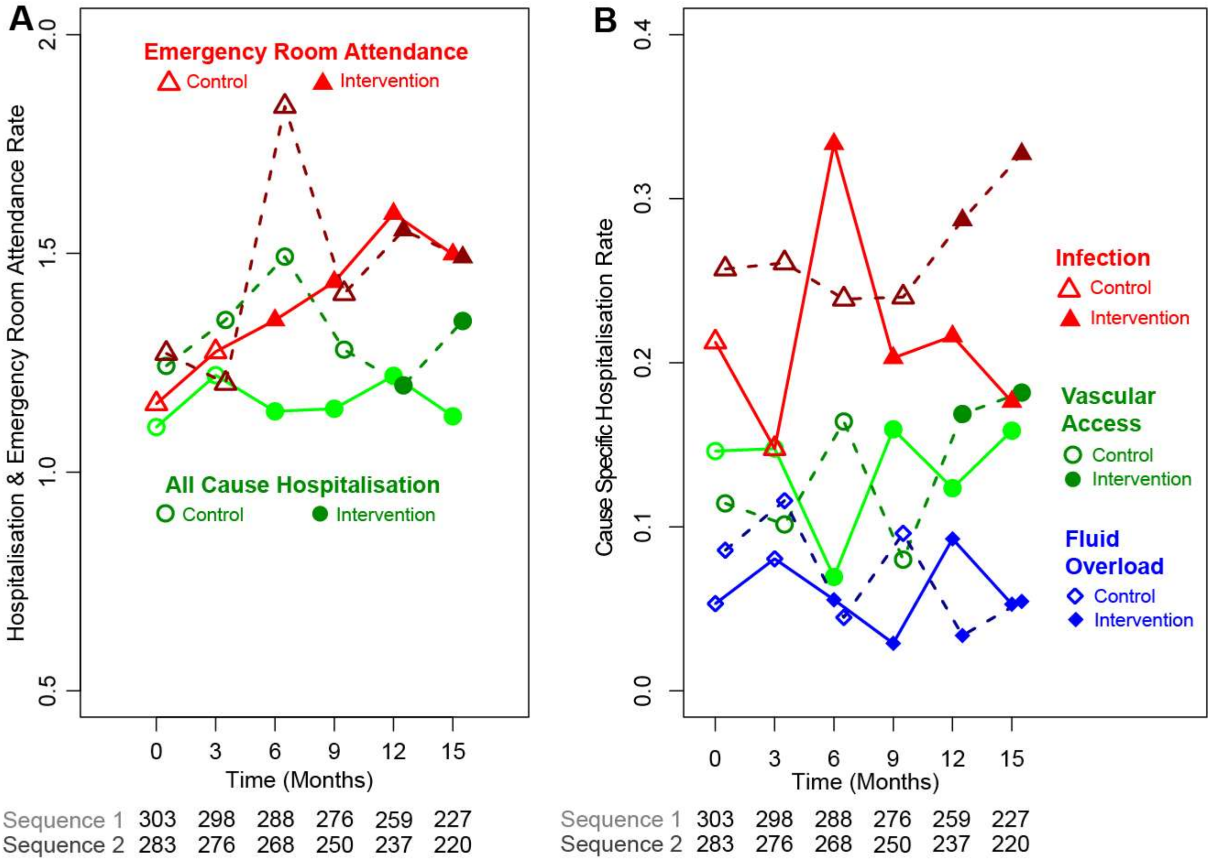

Supplement: S5 Fig — (PDF) [file pone.0253966.s006.pdf]
