# Supplementary material for: A breakthrough series collaborative to increase patient participation with hemodialysis tasks: A stepped wedge cluster randomised controlled trial
Source: PLoS One. 2021 Jul 20;16(7):e0253966. doi: 10.1371/journal.pone.0253966 (PMC8291659; doi:10.1371/journal.pone.0253966)
Supplement: S1 Table — (PDF) [file pone.0253966.s007.pdf]

S2 Table - Components of the breakthrough series collaborative underpinning the delivery of the intervention (adapted from[4])

	**Structure**	**Frequency**	**Attendees/Audience**	**Explanation**
Site Initiation Visit	Face to face at local Site	Prior to Learning Events	Programme representatives, trust clinical and quality improvement teams	Teams met in their own environment, and objectives of the programme presented. Understanding was gained of local team capability levels and programme expectations
Dialysis Task Data	Regular patient level data	Every 3 months	50 haemodialysis patients per site	Provided a common language and relevant patient data to discuss shared care and target interventions with teams, presented from transition period onwards to support targeted interventions and review progress
Learning Events	Whole day quality improvement learning events held centrally	2 events per quarter for a minimum of 5 events per wave.	All 12 trust teams in line with stepped wedge design. Minimum 5 team members including at least 1 patient representative per team.	To deliver a quality improvement curriculum, design and feedback on progress of local quality improvement activities and share learning including team building, motivation, patient stories etc.
Plan-Do-Study-Act (PDSA) cycles	Institute of Health Improvement Methodology	Designed at and executed after each learning event	Iterated locally and reported back to the collaborative.	To adapt the intervention at sites using data to enable local adoption – e.g. : to increase the number of pre and post dialysis weights recorded by a group of patients.
Action Period Call	Conference calls involving team members and programme leads.	Between each learning event.	All 12 Trusts Teams timed with the stepped wedge phases.	Opportunity to share progress with Plan Do Study Act cycles, enable teams to solve problems collaboratively, share experiences and learn from each other.
Patient partnership	Patient lead and network of patient representatives from each site.	Throughout programme	Participated in program board, all learning events, and evaluation board. Included patient break-out sessions at learning events that fed back the whole group.	In order to co-produce the intervention and ensure patient focus was maintained at sites.
Programme support including communication plan.	Site visits, telephone calls, email correspondence, news letters, website, social media.	Programme or site initiated as required	Programme and site team members as appropriate.	Maintain involvement, motivation, disseminate information, share progress and message of #whyidosharedcare and effect culture change. Twitter, WhatsApp, Facebook and the Shared HD Care Website[16]
Specific nurse training	Bespoke nurse training course available to participating teams	4 day course over 6 months available as required	Nurses from participating sites	Enabled health care staff to learn practical aspects of Shared Haemodialysis Care.
