# Supplementary material for: A breakthrough series collaborative to increase patient participation with hemodialysis tasks: A stepped wedge cluster randomised controlled trial
Source: PLoS One. 2021 Jul 20;16(7):e0253966. doi: 10.1371/journal.pone.0253966 (PMC8291659; doi:10.1371/journal.pone.0253966)
Supplement: S2 Table — (PDF) [file pone.0253966.s008.pdf]

S7 Table - ICD10 codes for cause-specific hospitalisation

Infection	A00-A33, A35-A99, D86, E321, E832, G00-G09, G374, G92, G937, H00-H10, H32, H660, H661, H662, H663, H664, H67, H70, H75, H830, H831, H921, H950, H951, I00-I02, I092, I32, I330, I39-I40, I41, I673, J00-J17, J181, J188, J189, J19-J21, J31-J32, J350, J36, J370, J371, J390, J391, J392, J40, J411, J47, J850, J851, J852, J86-J92, J94, J9502, K046, K047, K113, K122, K352, K353, K354, K355, K356, K357, K358, K359, K36-K37, K509, K518, K572, K610, K611, K612, K613, K614, K630, K65, K67-K68, K71, K750, K751, K752, K753, K758, K759, K764, K77, K81, K01-K08, L444, L702, L88, L928, L946, L980, L983, M00-M01, M021, M022, M023, M024, M025, M026, M027, M028, M352, M462, M463, M86, M908, N10-N12, N136, N151, N159, N16, N288, N300, N301, N302, N303, N308, N340, N341, N342, N343, N351, N37-N39, N41, N451, N452, N453, N454, N476, N481, N482, N49, N51, N61, N70-N74, N751, N760, N761, N762, N763, N764, N771, N980, O85, O861, O868, R091, R111, T802, T814, T826, T827, T835, T836, T845, T846, T847, T857, T874, T880, B00-B99
Fluid Overload	Z492, J969, J81, R060, J90, I50, E877, J989, I420, I13, J961, I313, R06, J91, J98, I10, I519, I139, J96, I11, R092
Vascular Access Complications	Z452, I770, I738, T824, T823, T828
