# Supplementary material for: A breakthrough series collaborative to increase patient participation with hemodialysis tasks: A stepped wedge cluster randomised controlled trial
Source: PLoS One. 2021 Jul 20;16(7):e0253966. doi: 10.1371/journal.pone.0253966 (PMC8291659; doi:10.1371/journal.pone.0253966)
Supplement: S3 Table — (PDF) [file pone.0253966.s009.pdf]

S8 Table
Individual symptoms of the POS-S Renal score (%)

Symptom (Moderate or worse)	Sequence 1	Sequence 2	Less than 5 tasks	5 or more tasks	n
Pain	39.5	37.8	40.9	36.4	485
Shortness of breath	31.5	34.2	31.8	33.9	485
Weakness	56.2	58.9	56.2	59.0	489
Nausea	18.6	15.1	18.2	15.7	488
Vomiting	13.7	8.0	10.8	11.4	487
Poor appetite	31.4	24.6	28.6	28.0	488
Constipation	17.6	17.0	16.7	18.5	484
Sore or dry mouth	27.4	21.3	20.9	28.7	488
Drowsiness	39.7	37.1	38.6	38.3	486
Poor mobility	52.6	44.0	53.6	42.5	489
Itching	40.2	36.0	40.1	36.2	486
Difficulty sleeping	47.0	43.6	44.2	46.7	489
Restless legs	40.2	32.1	36.1	37.4	485
Changes in skin	30.8	24.6	26.2	30.3	484
Diarrhoea	13.4	11.3	12.2	12.2	484
Anxiety	28.1	24.4	11.0	11.3	488
Depression	27.9	18.2	23.2	23.0	487
