# Supplementary material for: A breakthrough series collaborative to increase patient participation with hemodialysis tasks: A stepped wedge cluster randomised controlled trial
Source: PLoS One. 2021 Jul 20;16(7):e0253966. doi: 10.1371/journal.pone.0253966 (PMC8291659; doi:10.1371/journal.pone.0253966)
Supplement: S4 Table — (PDF) [file pone.0253966.s010.pdf]

S9 Table – Cluster (renal centre) characteristics

Cluster	1	2	3	4	5	6	7	8	9	10	11	12
n	63	43	27	51	47	52	32	40	48	50	80	53
Age (Years, mean (SD))	62.95 (16.53)	57.9 (14.19)	64.34 (15.41)	61.18 (16.87)	67.28 (15.1)	64.37 (13.99)	69.19 (14.64)	66.42 (14.95)	57.21 (16.26)	58.8 (14.34)	68.86 (13.24)	56.33 (15.45)
EQ5D Utility	0.73 (0.27)	0.79 (0.19)	0.66 (0.28)	0.66 (0.25)	0.77 (0.19)	0.63 (0.32)	0.76 (0.27)	0.76 (0.25)	0.72 (0.21)	0.69 (0.28)	0.59 (0.26)	0.65 (0.31)
Time on HD (Years, mean (SD))	5.4 (7.4)	3.6 (2.9)	6.1 (7.8)	8 (11.9)	7.1 (8.4)	5.9 (9)	4.8 (5.7)	3.9 (4.5)	4.8 (5.4)	4.8 (4.8)	6.1 (6.1)	5.1 (5.3)
Diabetes	0.433 (26/60)	0.538 (21/39)	0.409 (9/22)	0.195 (8/41)	0.39 (16/41)	0.5 (24/48)	0.5 (15/30)	0.257 (9/35)	0.378 (17/45)	0.417 (20/48)	0.506 (39/77)	0.261 (12/46)
Prev MI	0.183 (11/60)	0.205 (8/39)	0.364 (8/22)	0.146 (6/41)	0.098 (4/41)	0.125 (6/48)	0.3 (9/30)	0.114 (4/35)	0.244 (11/45)	0.208 (10/48)	0.247 (19/77)	0.13 (6/46)
Comorbid Score, mean (SD)	1.75 (1.47)	1.77 (1.44)	1.77 (1.48)	1.15 (1.57)	1.29 (1.23)	1.6 (1.27)	1.33 (0.88)	1.09 (1.5)	1.47 (1.55)	1.56 (1.24)	1.97 (1.49)	1.17 (1.14)
HHD Interest - Yes	0.133 (8/60)	0.238 (10/42)	0.111 (3/27)	0.167 (7/42)	0.15 (6/40)	0.149 (7/47)	0.188 (6/32)	0.105 (4/38)	0.261 (12/46)	0.146 (7/48)	0.063 (5/79)	0.133 (6/45)
No	0.8 (48/60)	0.548 (23/42)	0.889 (24/27)	0.643 (27/42)	0.65 (26/40)	0.66 (31/47)	0.719 (23/32)	0.816 (31/38)	0.609 (28/46)	0.708 (34/48)	0.861 (68/79)	0.4 (18/45)
Maybe	0.067 (4/60)	0.214 (9/42)	0 (0/27)	0.19 (8/42)	0.2 (8/40)	0.191 (9/47)	0.094 (3/32)	0.079 (3/38)	0.13 (6/46)	0.146 (7/48)	0.076 (6/79)	0.467 (21/45)
PAM score, mean (SD)	57.3 (20.4)	57.5 (10.9)	64.8 (20.7)	54.6 (13.3)	60.7 (27.7)	55.9 (15.9)	52.6 (17.5)	55.9 (16)	54.4 (11.6)	71.7 (21)	49.3 (17)	54.1 (17.2)
Tasks Independent & Supervised, mean (SD)	3.9 (3.9)	6 (4.2)	8.1 (3.5)	3.7 (2.4)	7.1 (3.5)	3.8 (3.7)	3.5 (1.8)	4.9 (3.2)	5.7 (3.7)	6.8 (2.7)	3.7 (3.5)	5.7 (6.4)
Kidney Transplant Centre	Yes	Yes	No	Yes	Yes	No	No	Yes	No	No	Yes	No
Prevalent dialysis population (n)	591	709	471	570	582	189	409	658	525	397	470	239
Home therapies/ Dialysis Ratio	0.19	0.08	0.14	0.14	0.13	0.21	0.25	0.16	0.07	0.27	0.24	0.09
Reported ICHD % non-white ethnicity	37%	54%	45%	24%	13%	5%	9%	14%	26%	35%	19%	5%
Recruited % non-white ethnicity	0.129 (8/62)	0.762 (32/42)	0.296 (8/27)	0.069 (2/29)	0.108 (4/37)	0 (0/37)	0.032 (1/31)	0.15 (6/40)	0.239 (11/46)	0.306 (15/49)	0.093 (7/75)	0.039 (2/51)

Adapted from Tabinor, M., A. Casula, M. Wilkie, S. Davies, F. Caskey and M. Lambie (2017). "UK Renal Registry 19th Annual Report: Chapter 13 Home Therapies in 2015: National and Centre-specific Analyses." Nephron 137 Suppl 1: 297-326.
