# Supplementary material for: A breakthrough series collaborative to increase patient participation with hemodialysis tasks: A stepped wedge cluster randomised controlled trial
Source: PLoS One. 2021 Jul 20;16(7):e0253966. doi: 10.1371/journal.pone.0253966 (PMC8291659; doi:10.1371/journal.pone.0253966)
Supplement: S5 Table — (PDF) [file pone.0253966.s011.pdf]

S10 Table – Subgroup analysis of primary endpoint

Endpoint Analysis	Time (per month)	Effect Size of Intervention (95% CI)	P	N
Low Activation Patients (n=130)				
Primary - 5+ Tasks or HHD				
Crude	1.02 (0.93 - 1.12)	1.83 (0.70 - 4.61)	0.218	130
Multivariable adjusted	1.01 (0.91 - 1.12)	2.22 (0.75 - 6.53)	0.148	108
Crude without time	-	2.23 (1.26 - 3.95)	0.006	130
Multivariable adjusted without time	-	2.38 (1.25 -4.52)	0.008	108
< 5 tasks at baseline (n=321)				
Primary - 5+ Tasks or HHD				
Crude	1.14 (1.07 - 1.22)	3.47 (1.73 - 6.98)	0.019	321
Multivariable adjusted	1.12 (1.04 - 1.21)	3.71 (1.66 - 8.31)	0.024	218
Crude without time	-	10.4 (6.42 - 16.92)	<0.001	321
Multivariable adjusted without time	-	9.43 (5.38 - 16.51)	<0.001	218
Poor Health Literacy (n=127)				
Primary - 5+ Tasks or HHD				
Crude	1.04 (0.95 - 1.14)	1.14 (0.45 - 2.89)	0.783	127
Multivariable adjusted	1.04 (0.95 - 1.15)	1.22 (0.44 - 3.34)	0.703	104
Crude without time	-	1.62 (0.94 - 2.80)	0.081	127
Multivariable adjusted without time	-	1.73 (0.95 - 3.13)	0.071	104
Incident Patients, <12 months HD (n=118)				
Primary - 5+ Tasks or HHD				
Crude	1.07 (0.97 - 1.18)	1.55 (0.57 - 4.25)	0.395	118
Multivariable adjusted	1.05 (0.94 - 1.16)	2.17 (0.70 - 6.67)	0.178	91
Crude without time	-	2.79 (1.52 - 5.14)	0.001	118
Multivariable adjusted without time	-	3.23 (1.62 - 6.43)	0.001	91

Adjusted for the baseline variables of age (categories), gender, time on dialysis (years), marital status, health literacy (adequate or inadequate), EQ5D utility value and comorbid score (derived from chronic obstructive pulmonary disease, congestive cardiac failure, cerebrovascular accident, acute myocardial infarction, neurological disease, vascular intervention, valvular heart disease, cancer, connective tissue disease and diabetes)
