# Supplementary material for: A breakthrough series collaborative to increase patient participation with hemodialysis tasks: A stepped wedge cluster randomised controlled trial
Source: PLoS One. 2021 Jul 20;16(7):e0253966. doi: 10.1371/journal.pone.0253966 (PMC8291659; doi:10.1371/journal.pone.0253966)
Supplement: S6 Table — (PDF) [file pone.0253966.s012.pdf]

**S11 Table – Hospitalisation rates, rate ratios and odds ratios**

| | Intervention | Control |
|---|---|---|
| All Cause Hospitalisation Rate (per annum) | 1.19 (1.08 - 1.31) | 1.28 (1.17 - 1.39) |
| Rate Ratio | 0.82 (0.91 - 1.06, P=0.282) | |
| Multivariable Odds Ratio | 1.00 (0.68 – 1.47, P=1.00) | |
| | | |
| Infection Hospitalisation Rate (per annum) | 0.26 (0.21 - 0.31) | 0.22 (0.18 - 0.28) |
| Rate Ratio | 1.15 (0.85 - 1.54, P=0.349) | |
| Multivariable Odds Ratio | 1.15 (0.62 – 2.11, P=0.662) | |
| | | |
| Fluid Overload Hospitalisation Rate (per annum) | 0.05 (0.03 - 0.08) | 0.08 (0.05 - 0.11) |
| Rate Ratio | 0.67 (0.37 - 1.21, P=0.162) | |
| Multivariable Odds Ratio | 0.24 (0.07 – 0.80, P=0.019) | |
| | | |
| Vascular Access Hospitalisation Rate (per annum) | 0.14 (0.11 - 0.18) | 0.13 (0.09 - 0.17) |
| Rate Ratio | 1.11 (0.74 - 1.66, P=0.592) | |
| Multivariable Odds Ratio | 0.76 (0.33 – 1.73, P=0.508) | |
| | | |
| Emergency Room Attendance Rate (per annum) | 1.48 (1.36 - 1.61) | 1.35 (1.24 - 1.47) |
| Rate Ratio | 1.1 (0.97 - 1.23, P=0.062) | |
| Multivariable Odds Ratio | 1.00 (0.70 – 1.42, P=0.985) | |

Adjusted for the baseline variables of age (categories), gender, time on dialysis (years), marital status, health literacy (adequate or inadequate), EQ5D utility value and comorbid score (derived from chronic obstructive pulmonary disease, congestive cardiac failure, cerebrovascular accident, acute myocardial infarction, neurological disease, vascular intervention, valvular heart disease, cancer, connective tissue disease and diabetes)
