# Supplementary material for: A breakthrough series collaborative to increase patient participation with hemodialysis tasks: A stepped wedge cluster randomised controlled trial
Source: PLoS One. 2021 Jul 20;16(7):e0253966. doi: 10.1371/journal.pone.0253966 (PMC8291659; doi:10.1371/journal.pone.0253966)
Supplement: S1 Text — (PDF) [file pone.0253966.s013.pdf]

S3 Text – Intervention Fidelity Assessment

Intervention fidelity was quantitatively assessed using an existing framework.[18] Delivery of the specified components of the breakthrough series collaborative and the conduct of PDSA cycles by sites were used to assess intervention content. The proportion of staff teams exposed to the intervention components and the proportion of patients asked about dialysis tasks as scheduled during the intervention assessed coverage and frequency of the intervention. Cluster and patient drop-out assessed the duration of intervention.

All nine planned learning events and thirteen action period calls were conducted as scheduled (supplementary figure S1).

The overall number of individual sites attendances at individual learning events was 75 of a possible 78 (two sites did not attend on a total of three occasions across three learning events) with an average of 4.14 team members per site per event (3.9 in sequence 1 and 4.6 in sequence 2). Ten of the twelve centres had representatives who participated in action period calls: 35 attendances of a possible 115 (30.4% overall, 30.1% in sequence 1 and 31.0% in sequence 2).

Eleven of the twelve sites conducted at least one Plan-Do-Study-Act cycle after their first learning event and reported it to the collaborative at the subsequent event (one site in sequence 2 chose a different quality improvement method).

91.9% (487/530) of patients who completed the task questionnaire during the baseline period that were still under follow-up when they transitioned into their intervention period completed the dialysis task questionnaire during this intervention period. Participation numbers at the time of moving from control to intervention were 290/303 (95.7%) in sequence 1 (transition in month 6) and 240/283 (84.8%) in sequence 2 (transition in month 12).
