# Supplementary material for: A breakthrough series collaborative to increase patient participation with hemodialysis tasks: A stepped wedge cluster randomised controlled trial
Source: PLoS One. 2021 Jul 20;16(7):e0253966. doi: 10.1371/journal.pone.0253966 (PMC8291659; doi:10.1371/journal.pone.0253966)
Supplement: S1 File — (PDF) [file pone.0253966.s014.pdf]

A quality improvement collaborative to scale up Shared Haemodialysis Care for patients on centre based haemodialysis.

Short Title / Acronym: SHAREHD
IRAS project ID : 212395
Trial Sponsor : Sheffield Teaching Hospital NHS Foundation Trust
STH research number : 18999
Funding Source: Health Foundation.

1. Project details

Investigator details

Chief Investigator for SHAREHD	Professor Martin Wilkie<br>Consultant Nephrologist<br>Sheffield Kidney Institute<br>Sheffield Teaching Hospital NHS Foundation Trust<br>Herries Road<br>Sheffield S5 7AU<br>martin.wilkie@sth.nhs.uk<br>T +44 (0) 114 271 5326
Study Sponsorship	R & D Contact<br>Dr Dipak Patel,<br>Research Manager, Clinical Research Office Sheffield<br>Sheffield Teaching Hospitals NHS Foundation Trust<br>D Floor, Royal Hallamshire Hospital<br>Glossop Road, Sheffield S10 2JF<br>Dipak.Patel@sth.nhs.uk<br>T +44 (0) 114 2265941 Fax. +44 (0) 114 2265937<br>http://www.sheffieldclinicalresearch.org
Project title	A quality improvement collaborative to scale up Shared Haemodialysis Care for patients on centre based haemodialysis : SHAREHD
STH Project reference number	STH 18999
Protocol version number and date	Draft V1.3, date 27th July 2016
Funder	Health Foundation – award reference number 7664 June 2016
STH Directorate affiliation	Renal academic directorate
NIHR CRN number	

Signatures

Chief Investigator	
Date	
R & D contact	
Date	

Collaborating hospital sites

Hospital site	Principal investigator
Central Manchester Health Care Trust	Dr Sandip Mitra
City Hospitals Sunderland NHS FT	Dr Saeed Ahmed
East & North Hertfordshire NHS Trust	Dr Paul Warwicker
Guy's & St Thomas NHS Foundation Trust	Dr Nicola Kumar
Heart of England Foundation Trust	Dr Joyti Baharani
Leeds Teaching hospitals NHS Trust	Dr Elizabeth Garthwaite
North Bristol NHS Trust	Dr Albert Power
Nottingham University Hospitals NHS Trust	Dr Alastair Ferraro
Sheffield Teaching Hospitals NHS Foundation Trust	Dr Veena Reddy
The Royal Wolverhampton NHS Trust	Dr Johan Nicholas
University Hospital of North Midlands	Dr Mark Lambie
York Teaching Hospitals Foundation Trust	Dr Paul Laboi

Collaborating partners

Yorkshire and Humber CLAHRC, University of Sheffield.

Dr Steve Ariss, Dr Tracey Young, Prof Stephen Walters.

UK Renal Registry Transforming Participation in Chronic Kidney Disease Programme.

Ron Cullen, Dr Fergus Caskey, Rachel Gair.

1. Project details 1
Investigator details 1
Signatures 1
Collaborating hospital sites 2
Collaborating partners 2
2. Research question 5
3. Abstract 5
4. Aim of the study 5
5. Background 5
6. Plan of investigation 6
Methodology 6
Intervention 6
Study design 7
Setting 7
Participants 7
Inclusion 7
Exclusion 7
Endpoints 7
Efficacy Endpoints 7
Quality and Safety Endpoints 8
Evaluation Endpoints 8
Recruitment 8
Data Collection 8
Patient Questionnaires 8
Routine Data 8
Patient Interviews: 9
Staff interviews 9
Analysis including statistical methods 10
Statistical analysis: 10
Economic Evaluation (Efficiency) 10
Method 10
7. Statistical opinion 11
Sample size: Power of the study. 11
8. Project management 11
9. Expertise 11

10. Governance, ethics and confidentiality 12
Information Governance and Data Linkage 12
Patient information sheet and consent forms 14
11. Service users: involvement during the study design 14
12. Dissemination. 14
13. Taking the work forward. 15
14. Intellectual property. 15
15. Costing schedule 15
16. Funding arrangements 16
17. Abbreviations 16
18. List of attachments to this protocol 16
19. References 16

2. Research question

This protocol describes data collection, database linkage and qualitative evaluation necessary to answer the following research questions -

> Primary question: To what extent does a programme of structured learning increase patient participation in haemodialysis activities or result in independent haemodialysis?
>
> Secondary question (a): What are the economic consequences of introducing a programme of structured learning to increase patient involvement in haemodialysis?
>
> Secondary question (b): In terms of encouraging patient involvement in haemodialysis, what works, for whom, in what circumstances and why?

3. Abstract

This 24 month research study is nested within a 30 month quality improvement project that aims to scale up Shared Haemodialysis Care (SHC) to 12 dialysis centres across England. SHC is where patients who receive haemodialysis (HD) at dialysis centres are given the opportunity to learn some of the tasks associated with their treatment.

This enhanced person centred care is intended to improve the experience for those who choose to dialyse at hospital, and give more patients the confidence to select home dialysis leading to a better quality of life, aligning with NICE guidance(1).

Following a 6-month set up period, a phased implementation programme will commence across a minimum of 12 dialysis units using a randomised stepped wedge design. During these stepped phases the following measurements will be obtained through patient questionnaires and through linkage with routinely collected data at the end of the study:

- process - the number (%) of patients performing at least 5 tasks collected using 3 monthly questionnaires.
- outcome – number (%) of people choosing to perform home haemodialysis (HHD) or dialyse independently in-centre, measures of patient experience.
- balancing – end-user recommendation; home dialysis establishment delay; staff impact confidence survey; hospitalisation and infection.
- Costs – health economics of shared haemodialysis care.

The research database arising from this evaluation will be used to explore hypothetical barriers to patient and centre level adoption of SHAREHD with a view to developing future interventions. Running parallel with the stepped wedge assessment of SHAREHD we will develop a quality improvement collaborative to refine the intervention for local adoption.

4. Aim of the study

The study aims to assess the effectiveness and economic impact of a structured programme to encourage patient involvement in haemodialysis, and to understand what work for whom in what circumstances and why.

5. Background

Renal failure has a major impact on quality of life and survival. Haemodialysis is the most common form of dialysis and involves the patient's blood passing through a filter to remove toxins using a machine. In England approximately 20,000 people attend institutions three times (totalling approximately 16 hours) per week to receive HD. Most are passive recipients of their care, engaging little with their own treatment. In contrast, patients who perform HHD have better survival (2) and

quality of life (3), as well as the treatment being more cost-effective (4). This is important since NHS healthcare costs for patients with renal failure are high (£636M per year), consuming approximately 2% of the NHS budget for 0.05% of the patient population. Currently only 4.1% of patients dialyse at home (5), with considerable variation between centres in the UK (0 to 11.5%) and internationally (average 12.9% in Australia and New Zealand) (6). Increasing HHD in the UK to international levels would lead to estimated annual savings to the NHS of £16M (estimating an increase of 1400 people treated with HHD at a saving of £12000 per patient per year(7)).

There is considerable evidence of the benefits of supported self-care in long term conditions (8, 9). Low health literacy amongst dialysis patients is associated with worse survival (10) whereas self-motivation and patient education results in better care e.g. phosphate control (11) and fluid balance (12). As with the broader NHS, dialysis services are experiencing considerable pressure to deliver high quality in the face of fiscal challenge. An important mechanism to ensure that quality of care is maintained is to engage service users as true partners in their own care; self-management is an ambition in "Kidney Health: Delivering Excellence"(13). Although dialysis is a technical treatment, it can be performed by patients with suitable training and support. Indeed renal patients have a long tradition of self-care, for example 1,113 UK patients performed haemodialysis at home in 2013 (1). However, the likelihood of undertaking home dialysis is influenced by several factors including physician enthusiasm, area deprivation and ethnicity(14).

Shared Haemodialysis Care describes an intervention where people treated with in-centre haemodialysis are given the opportunity to learn tasks relating to their own dialysis treatment. HD treatment is standardised requiring a number of key steps for its preparation, delivery and discontinuation. The 14 tasks forming part of the treatment include the measurement of observations (weight, blood pressure); infection control (washing hands and dialysis access sites); preparation of the machine; establishing dialysis access; running the dialysis; discontinuing treatment; disconnecting access and clearing away. To achieve this, health care professionals need to enhance their roles, becoming educators and facilitators, supporting patients to take a greater role in their own care. This gives centre based dialysis patients access to the benefits of engaging in their own care while increasing the opportunities for dialysing at home.

6. Plan of investigation

Methodology

The objective of this study is to assess the impact of the SHAREHD intervention across a range of domains. Through the study we will collect evidence to demonstrate the effectiveness of the intervention and explore hypothetical associations between patient and organisational characteristics, shared HD uptake, its impact on the design of dialysis services and outcomes. To achieve this we will undertake a 24 month stepped-wedge implementation at 12 dialysis centres in England.

Intervention

The intervention is where a trained dialysis nurse asks the patient which haemodialysis related treatment tasks they would like to learn to do. This intervention is supported by nurse education, patient and carer training, supportive materials and clearly defined competencies. The quality improvement (QI) collaborative will utilise learning packages to support the implementation and measurement of the intervention.

Study design

A stepped-wedge intervention in which 6 centres will be randomly allocated to start implementation in phase 1, with the remaining 6 starting in phase 2. The study phases are outlined in table 1 and the schedule of activities in table 2.

Table 1 – study phases.

Phase	Start point	Action
Baseline	Month 0	Commence data collection
Phase 1	Month 6	Start implementation with first 6 centres
Phase 2	Month 12	Start implementation with second 6 centres
Sustainability	Month 18	Data analysis, report writing, business case development

Table 2 – schedule of study activities

	Months of study							
	baseline		Step 1		Step 2		Sustainability	
**Activity**	**0**	**3**	**6**	**9**	**12**	**15**	**18**	**21**
Patient identification	X							
Informed consent	X							
Demographic Questionnaire	X							
Task summary sheet	X	X	X	X	X	X	X	X
Composite questionnaire	X		X		X		X	
Patient appreciation card			X		X		X	
Status change form	Event driven							
Patient / carer interviews & workshops	48 interviews with 24 patients throughout the study period							

Setting

12 dialysis centres in England selected on the basis of organisational characteristics and variation in demographics and utilisation of home dialysis.

Participants

The assessment of SHAREHD uptake and patient level impact will be formally undertaken in a core sample of 600 patients sequentially selected from within 12 dialysis centres.

Inclusion

- Established on centre based haemodialysis
- Capacity to give written informed consent to participate in the study

Exclusion

- Patients who are too unwell to engage in the study, as judged by the clinical team
- Patients unable to understand written and verbal communication in English

Endpoints

Efficacy Endpoints

The primary binary outcome is whether or not haemodialysis patients can learn and complete 5 or more out of 14 tasks.

The secondary binary outcome is an increase in home haemodialysis and in centre independent dialysis of 4% within participating centres

Quality and Safety Endpoints

Changes in patient activation, quality of life, renal-specific symptoms and hospitalisation (all-cause and cause specific).

Evaluation Endpoints

Cost effectiveness

Recruitment

All suitable prevalent dialysis patients at selected centres will be entered into a screening log and from this log patients will be approached and given the opportunity to participate in the research with the intention of recruiting up to 50 patients at each site. As the consent is simply for permission to collect data we expect to be able to recruit more than 90% of eligible patients at each centre. Informed consent will be taken by trained delegated members of the local research team who have appropriate Good Clinical Practice certification. Patients will be given the information sheet at a dialysis session to read and take home. At the subsequent dialysis session consent will be taken after giving the opportunity to ask questions based on the information sheet. Participants will also be given the opportunity to participate in interviews and workshops conducted by the evaluation team. The detail of these is discussed under Patient Interviews. A consent schema is presented on page 14.

Data Collection

Patient Questionnaires

We will collect measures as outlined in table 2. Measures will be collected by a delegated member of the local research team by asking patients to complete data forms as detailed in the patient information sheet and in table 3. These questionnaires will be administered while waiting for or in the early part of the patient's dialysis session.

Table 3 – Measures and their frequencies during the cohort study

Measure	Frequency	Collection method
Demography form[1]	Once, at start	Paper form
Tasks summary sheet	3 monthly	Paper form
Composite questionnaire including : Your Health Survey, SHAREHD uptake indicators form and the Health Economic questionnaire	6 monthly	Paper form
Status change form	Event Driven	Paper form

1 – Adapted from the Yorkshire dialysis decision aid study (15).

Routine Data

Admissions to hospital, both before the start of the SHAREHD programme and following the implementation will be obtained through linkage to the Hospital Episode Statistics (HES) database. HES will be used for information on comorbidity at the time of recruitment (by reviewing diagnosis codes from admissions up to 5 years prior to the start of the SHAREHD programme), hospitalisation (all cause and cause specific) before and after the implementation of SHAREHD to assess for benefits and harms, and inform health economic analyses. Members of the research team have previously

used these information sources to assess costs and comorbidity. Similar datasets and diagnostic codes have been used to identify harms from hospital and home based haemodialysis therapies.

The UK Renal Registry (UKRR) will provide patient level data relating to dialysis quality, recognised audit standards and the uptake of home dialysis therapies in the participating centres, as part of core analysis which informs their annual report.

Patient Interviews:

We will conduct semi-structured interviews, based on a realist framework with approximately 24 patients in total (an average of two at each site), or until we are satisfied that data saturation has been reached. These patients will be followed up to provide interview data at two time points: before and after the implementation of the shared care programme (approximately 48 interviews). The interviews will be focused on developing, testing and refining specific hypotheses that are considered important for understanding what is working, for whom, in what circumstances and why.

Initially, a maximum variation sampling strategy will be followed. However, the sampling framework might be subject to change, as this will be defined by the development of the programme theory. The initial sampling framework will be based on key characteristics of the participating sites and individual service-users, which are considered important in determining variability in implementation and outcomes.

Potential respondents will be identified during the consent process for the data linkage/collection activities. The consent form for these research activities will contain an item for service-users to indicate whether they would be happy to be approached for an interview and a space to provide contact details. This will potentially provide a large pool of possible respondents that can be drawn upon depending on the developing sampling criteria.

Interviews will be carried out at an appropriate location (for instance a room at the participating Trust, or at the patient's home). These will last approximately 45-60 minutes and be audio recorded. The recordings will be transferred to an encrypted storage device as soon as possible and identified with a code. These recordings will be transcribed verbatim, and then the audio recordings will be destroyed. Transcripts will be anonymised to remove any information, which might identify the respondent, and stored as described below (section 10).

Transcripts will be entered into NVIVO qualitative data analysis software to aid data management analysis and retrieval, and analysed in order to identify what works, for whom, under what circumstances and why. Anonymised sections of transcripts will be used to support the development and refinement of hypotheses in study outputs.

Patient workshop groups:

Approximately six workshops with patients will be conducted over the course of the project. These will be intended to present emerging findings to service-users and validate findings in a group setting. They will be used to rapidly investigate service-user perspectives and investigate convergence and divergence of experiences and opinions. They will last a maximum of two hours, and contemporaneous notes will be taken and they will be audio recorded.

Staff interviews

We will also conduct interviews with key stakeholders involved in the provision and commissioning of services. However, these activities do not fall under the remit of NHS ethics procedures, and we will seek ethics review for these activities through the University of Sheffield ethics review process.

Analysis including statistical methods

Statistical analysis:

The primary binary outcome is whether or not HD patients can learn and complete 5 or more out of 14 tasks. This outcome will be collected on paper forms for the 12 participating renal units over three, six-month duration, time periods or steps (Step 1 (baseline): 0 to 6 months, Step 2: 6 to 12 months; Step 3: 12 to 18 months. This binary outcome will be compared across the intervention and control clusters using a random (or multi-level mixed) effects logistic regression model with time (or step), group (intervention or control) and individual patient characteristics such as age and sex as covariates; and the renal unit or cluster as a random effect. This model will take into account the clustering of outcomes by renal units. The odds ratio estimate for the intervention effect and its associated confidence interval will be reported from the model.

Statistical associations between patient characteristics, dialysis schedules and outcomes will also be explored.

Economic Evaluation (Efficiency)

The economic evaluation will take an NHS and social care perspective and will compare SHC with usual care in a cost-utility analysis using a cost-per quality adjusted life years (QALYs) approach. We will also provide a cost effectiveness analysis to estimate resources required for changes in primary and secondary outcomes (number of tasks performed and number of patients performing independent dialysis).

Method

The EQ-5D-5L will be used to measure health related quality of life at baseline, six and 12 months. The costs of the intervention will be collected throughout the study and will include set-up and running costs such as training, room hire, refreshments, materials, staff time, web site and staff.

Resource use will be estimated from a range of sources including questionnaires, observation and organisational documentation. A resources use questionnaire will be based on questionnaires from the database of instruments for resource use measurement. Unit costs, derived from appropriate national sources will include; NHS reference costs, Personal Social Service Research Unit (PSSRU) costs, and the Office of National Statistics.

We will use an adaptation of the annotated cost questionnaire[1] in order to capture information from patients on time and distance travelled, total time spent between pick up for dialysis and drop off, whether a companion accompanied/spent time with them during a haemodialysis session (home or hospital) and whether arrangements had to be made for any dependents when attending a session.

Observation: In order to establish the differences in time required for traditional dialysis, shared dialysis and independent unit-based dialysis, non-participant observation will be carried out. The researcher will attend the dialysis unit and use an observation template to record activities. The purpose of these observations is to identify any changes in processes and how much time is taken for specific activities. Patient identifiable data will not be collected.

---

[1] Thompson S & Wordsworth S on behalf of the UK working party on patient costs. An annotated cost questionnaire for completion by patients. HERU Discussion Paper No [03/01]

Organisational documentation: In order to assess service-level economic performance, routinely collected data, such as numbers of patients being treated and levels of staffing will be gathered through study coordinators/champions based at each site.

7. Statistical opinion

Sample size: Power of the study.

The primary binary outcome is whether or not HD patients can learn and complete 5 or more out of 14 tasks that form part of their own dialysis treatment. If we assume that the baseline level is around 15%, an ICC of 0.05 and an average cluster size of 25 HD patients; then using the STATA stepped wedge command (16) with a stepped wedge design of 3 steps (including baseline) and 12 clusters, with 6 clusters randomised at each step will we have 90% power to detect an increase in the event rate from 15% to 30% as statistically significant at the 5% two-sided level.

A secondary outcome is the number and proportion of HD patients who have Home HD or dialyse in centre independently. If we assume that the baseline level is around 2% in our clusters an ICC of 0.05 and an average cluster size of 25 HD patients; then with a stepped wedge design of 3 steps (including baseline) and 12 clusters, with 6 clusters randomised at each step will we have 80% power to detect an increase in the event rate from 2% to 7.2% as statistically significant at the 5% two-sided level.

8. Project management

Study oversight will be an evaluation advisory board that will review study progress and supervise the return of appropriate data to sites so that it can be used in clinical consultation. The study will have a dedicated project manager to ensure that data collection is timely and of high quality.

9. Expertise

The study group has expertise in several areas –

Martin Wilkie has lead the Health Foundation and Kidney Research UK funded Yorkshire and Humber Shared Haemodialysis Care initiative during which the current concepts were developed and is Chief Investigator for the UK Catheter Study (a questionnaire related cohort study of peritoneal dialysis access)

Andy Henwood - Renal patient with direct experience of SHC. Andy has a training and development background, and was employed by the Y&H SHC to co-develop the training course. He sits on the Y&H Renal Network Strategy Group.

Steven Ariss is a Senior Research Fellow in Health Service Research at the University of Sheffield. He has expertise in complex evaluation and a wide range of qualitative and quantitative methodologies. He is experienced in leading large multi-centre programmes. He holds influential posts in the health care innovation infrastructure including Evaluation Lead for the NIHR CLAHRC for Y&H. He is the Module lead for 'Complex Evaluation Methods' (Masters in Clinical Research). As the Innovation and Knowledge Translation Lead for Health Services Research at the University of Sheffield, he also provides evaluation consultancy services for a range of NHS, third sector and private organisations.

Stephen Walters is a Chartered Statistician with over 20 years' experience of the design, analysis and reporting of trials of complex interventions to capture the effectiveness and cost-effectiveness of a variety of health technologies. He is a Fellow of the Royal Statistical Society and member of the NIHR HTA Clinical Evaluation and Trials board and the Irish Health Research Board.

Tracey Young is a Health Economist with over 20 years' experience in health service research; she has developed methodology for developing health economic outcome measures. Tracey has experience of economic evaluations in trials and observational studies (e.g. liver/small bowel transplantation and aphasia), and has worked with large datasets including TARN and HES. She is a Member of the Health Economics and Decision Modelling section in ScHARR, which has an international reputation in developing and application of methodology. Tracey is the Acting Lead for the health economic and outcome measurement theme in the NIHR CLAHRC for Y&H.

James Fotheringham has led previous data linkage programmes, data analysis and evaluation of cost in ESRD patients. He is a member of the NIHR Haemodialysis study group, and has ongoing collaboration in other projects with UKRR and KRUK. He will lead on clinical data linkage and provide specialist clinical advice and guidance.

Joanna Blackburn is a research fellow working in the Research and Development department at Barnsley Hospital and holds an honorary contract with the Public Health section in ScHARR. She has been involved in a number of service evaluations (e.g. Diabetes and Emergency Department) and has coordinated clinical trials. She has experience of conducting qualitative research and has worked as a researcher on several projects in the Healthy Weight theme in the NIHR CLAHRC for Y&H.

Tania Barnes was involved in developing national nurse training course for Shared Haemodialysis Care and organises and delivers that course. She has considerable experience in the nursing aspects of SHC.

Dr Sandip Mitra is a consultant nephrologist with particular experience in home haemodialysis and the analysis of gaps in unmet needs in technology in relation to SHC with particular consideration of the patient perspective.

Rachel Gair Leads Transforming Participation in CKD Programme and has experience as Renal Network Manager for the South West. Her skills include programme management, quality improvement and communications.

Paul Harriman is Assistant Director of quality improvement at STH and his interests include system thinking, improvement science, use of data for decision making.

Michael Nation (governance lead) is the development director Kidney Research UK and has extensive experience (>10 years) of large scale and multi-site site collaborative project development, implementation and delivery, including three QI projects.

10. Governance, ethics and confidentiality

NRES review will be undertaken before the study proceeds for all work taking place in the NHS regarding the collection and use of identifiable patient data, or contact with patients. All participants will sign informed consent forms after reviewing a patient information sheet and taking time to consider whether to participate.

Information Governance and Data Linkage

Paper questionnaires will be batched, stored securely at local sites and securely transferred within 1 month of collection by courier to the Sponsor site (Sheffield Teaching Hospitals NHS Trust) to be entered into a bespoke, password protected, research database held on a secure server with limited access within the trust. Paper questionnaires will be retained in secure rooms, in locked cabinets at STH for audit purposes. Qualitative data will be pseudo anonymised at source and any identifying data removed during transcription. Audio recordings will be destroyed after transcription. A securely-stored, separate key will be used to link data with participants' identifying data (such as

name and contact details). Study participants will be given a unique identification number for the purposes of the study. The number will include centre number, patient initials and a sequential patient number at that centre.

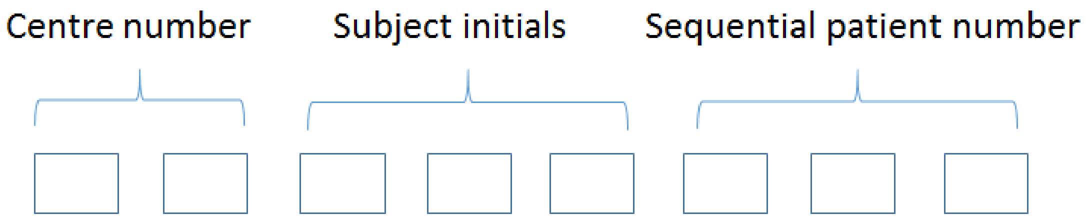

During the analysis phase, the research database including patient identifiable information will be transferred with the NHS number to the Health and Social Care Information Centre (HSCIC) Data Access Request Service (DARS). Currently this DARS uses the NHS Secure File Transfer system, ensuring that patient information can move securely between two NHS locations. Within their secure environment HES data will be linked to the research database and patient identifiers removed leaving a pseudo-anonymisation key to relate the various data items to an anonymised individual within the database. This pseudo-anonymised data will then be transferred back to SKI and the University of Sheffield for analyses detailed above, along with other secondary analyses which will be detailed in the DARS application.

All digital data will be stored in secure restricted access network locations within Sheffield Teaching Hospitals and the University of Sheffield. Movement of data between sites will be minimised, involve the movement of anonymised data where possible, and be carried out using encrypted digital storage devices.

<u>Diagram to describe secure data flow throughout the SHAREHD study.</u>

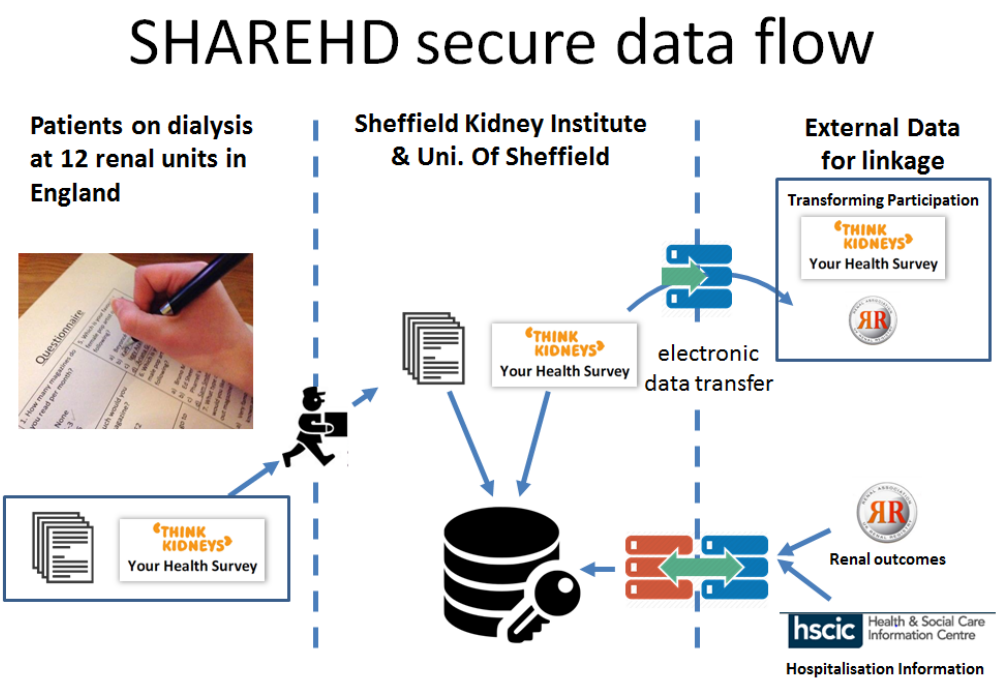

Data from the questionnaires will be inputted into the secure database held in Sheffield and the questionnaires will be destroyed after that. The data from "Your Health Survey" will be returned electronically and securely to the UK Renal Registry and from there individual information will be entered into Patient View so that it will be for participants to access themselves using secure passwords. The secure database will be destroyed on 31st December 2024. During the course of the study questionnaires may be re-formatted to permit automated data entry using an electronic scanner.

Patient information sheet and consent forms

These documents will describe the study and the uses that data is being put to including the linkage with Hospital Episode Summary Data, as well as sharing with the UK Renal Registry. The process of patient consent for both parts of the study is detailed below. Patients will be initially approached to participate in the cohort study that involves questionnaires and data linkage and from there will be given the opportunity to consent to participate in interviews and workshops.

Flow for consenting process - at each stage the participant has the option to decline

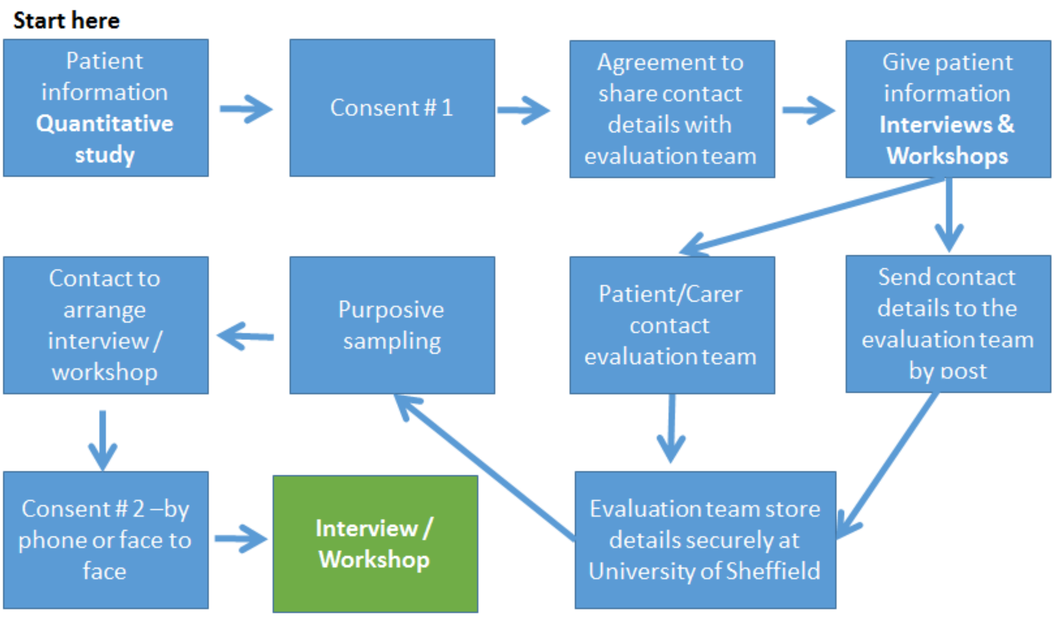

11. Service users: involvement during the study design

Patients have been involved throughout the design of the study and are represented on our project board. They will actively contribute to the co-design of the implementation pathway and the best way of collecting required data. A patient and user work-stream has been established to provide oversight of the study as it progresses. Information from the study will be returned to patients in a variety of formats including making key information available so that it can be used within clinical consultations. We will provide participants with appreciation cards during the study.

12. Dissemination.

We will utilise the learning from this research to feed back to site and under pin the quality improvement work that we are undertaking to Scale Up Shared Haemodialysis Care.

Our dissemination and communication will be through the following pathways:

Presentations at learning events, local, national and international meetings (patient, renal, QI, health service management)

Publish material for BJRM, Nursing Standard and various patient-facing publications circulated by our charity partners (below).

Patient focused material will be generated and disseminated by our national charity partners – including Kidney Research UK, BKPA and NKF, supported by NHS England. KRUK has considerable experience in delivering publicity campaigns and will co-ordinate the accreditation of outputs (such as training materials).

Key outputs will include -

- Educational and informational materials
- Training tool kit (aiming for NICE endorsement)
- Ongoing network supported by our knowledge sharing platform
- Implementation tool kit for organisations including business case template
- Peer reviewed publications

13. Taking the work forward.

Sustainability will be planned from the outset utilising documented best practice (1) including:

- Developing a knowledge sharing platform to strengthen networking, and providing access to http://www.shareddialysis-care.org.uk/ which supports patients, carers and staff members, hosting patient stories, data and discussion.
- Making local evidence of implementation and patient experience information available to participating units promptly, and incorporating this information into learning events.
- Linking in with Transforming Participation in CKD to sustain a patient peer support programme (2).
- Peer Assist methodology will be used to provide support from centres that already have experience with SHC to support patients and nurses.
- Learning events held annually to provide an opportunity to share experience and review results.

14. Intellectual property.

The study team will liaise with the Health Foundation (HF) regarding intellectual property, will recognise the HF in all communications and publications coming out of this work, and share such outputs in advance with the HF to allow time for comment. Advice on this aspect will be sought from Medipex (www.medipex.co.uk).

15. Costing schedule

The study costs are as set out in the table 4 below. Research nursing costs at £20 per hour.

Table 4 – study costs.

Study event	Type of cost	Staff time per item	Number	Total cost
Patient eligibility, approach and consent	Support cost	20 mins	600	CRN
Demography*	Research	5 mins	600	£1000
Task questionnaire*	Research	5 mins	600 x 8	£8000

	cost			
Composite questionnaire (includes Your Health Survey, Uptake indicators form and Health Economic Questionnaire) *	Research cost	9 mins	600 x 4	£4000
Status Change questionnaire	Research Cost	120 mins per site		£480
Batching & courier costs per site	£20 per courier	£100 per site x 11		£1100
Data linkage HSCIC~				£1435
**total**				**£16015**

*To be completed by the patient and administered by the local research team and sent back to the central site.
~Data linkage and extract service charges HSCIC 2015/16 (included in the Health Foundation grant.

16. Funding arrangements

Funds will be utilised from the Health Foundation Scaling Up Award. Site payments will be made on 6 monthly receipts of validated data.

17. Abbreviations

CLAHRC : Collaboration for Leadership in Applied Health Research and Care
HHD : home haemodialysis
HSCIC : Health and Social Care Information Centre
NRES : National Research Ethics Service
NIHR : National Institute for Health Research
QI : quality improvement
ScHARR : Sheffield School of Health and Related Research
SHC : shared haemodialysis care
STH : Sheffield Teaching Hospitals NHS Foundation Trust
UKRR : UK Renal Registry
UoS : University of Sheffield

18. List of attachments to this protocol

a) Patient information sheet and consent form cohort study (quantitative)
b) Patient information sheet and consent form – interviews and workshops
c) Status change form
d) Demography form
e) Health economic questionnaire
f) Patient appreciation text
g) Patient interview questions
h) Uptake indicators form
i) Workshop plan
j) Tasks summary sheet
k) Your health survey

19. References

1. NICE. Guidance on home compared with hospital haemodialysis for patients with end-stage renal failure. 2002.

2. Nitsch D, Steenkamp R, Tomson CR, Roderick P, Ansell D, MacGregor MS. Outcomes in patients on home haemodialysis in England and Wales, 1997-2005: a comparative cohort analysis. Nephrol Dial Transplant. 2011;26(5):1670-7.
3. Watanabe Y, Ohno Y, Inoue T, Takane H, Okada H, Suzuki H. Home hemodialysis and conventional in-center hemodialysis in Japan: a comparison of health-related quality of life. Hemodial Int. 2014;18 Suppl 1:S32-8.
4. NICE. Guidance on home compared with hospital haemodialysis for patients with end-stage renal failure. 2002. Report No.
5. Shaw C, Pitcher D, Pruthi R, Fogarty D. UK Renal Registry 16th annual report: chapter 2 UK RRT prevalence in 2012: national and centre-specific analyses. Nephron Clin Pract. 2013;125(1-4):29-53.
6. Agar JW, Hawley CM, Kerr PG. Home hemodialysis in Australia and New Zealand: how and why it has been successful. Semin Dial. 2011;24(6):658-63.
7. Baboolal K, McEwan P, Sondhi S, Spiewanowski P, Wechowski J, Wilson K. The cost of renal dialysis in a UK setting--a multicentre study. Nephrol Dial Transplant. 2008;23(6):1982-9.
8. Elliott J, Rankin D, Jacques RM, Lawton J, Emery CJ, Campbell MJ, et al. A cluster randomized controlled non-inferiority trial of 5-day Dose Adjustment for Normal Eating (DAFNE) training delivered over 1 week versus 5-day DAFNE training delivered over 5 weeks: the DAFNE 5 x 1-day trial. Diabet Med. 2015;32(3):391-8.
9. McManus RJ, Mant J, Haque MS, Bray EP, Bryan S, Greenfield SM, et al. Effect of self-monitoring and medication self-titration on systolic blood pressure in hypertensive patients at high risk of cardiovascular disease: the TASMIN-SR randomized clinical trial. JAMA. 2014;312(8):799-808.
10. Cavanaugh KL, Wingard RL, Hakim RM, Eden S, Shintani A, Wallston KA, et al. Low health literacy associates with increased mortality in ESRD. J Am Soc Nephrol. 2010;21(11):1979-85.
11. Umeukeje EM, Merighi JR, Browne T, Victoroff JN, Umanath K, Lewis JB, et al. Self-Motivation Is Associated With Phosphorus Control in End-Stage Renal Disease. Journal of renal nutrition : the official journal of the Council on Renal Nutrition of the National Kidney Foundation. 2015.
12. Smith K, Coston M, Glock K, Elasy TA, Wallston KA, Ikizler TA, et al. Patient perspectives on fluid management in chronic hemodialysis. Journal of renal nutrition : the official journal of the Council on Renal Nutrition of the National Kidney Foundation. 2010;20(5):334-41.
13. Group KHA. Kidney Health: Delivering Excellence. info@britishrenal.org, 2013.
14. Castledine CI, Gilg JA, Rogers C, Ben-Shlomo Y, Caskey FJ. Renal centre characteristics and physician practice patterns * associated with home dialysis use. Nephrol Dial Transplant. 2013.
15. Winterbottom AE, Gavaruzzi T, Mooney A, Wilkie M, Davies SJ, Crane D, et al. Patient Acceptability of the Yorkshire Dialysis Decision Aid (Yodda) Booklet: A Prospective Non-Randomized Comparison Study across 6 Predialysis Services. Perit Dial Int. 2015.
16. K H, A G. A menu-driven facility for power and detectable-difference calculations in stepped-wedge cluster-randomized trials. The Stata Journal. 2014;14(2):363-80.
